# Longitudinal follow up of a boy affected by Pol III-related leukodystrophy: a detailed phenotype description

**DOI:** 10.1186/s12881-015-0203-0

**Published:** 2015-07-25

**Authors:** Roberta Battini, Silvano Bertelloni, Guja Astrea, Manuela Casarano, Lorena Travaglini, Giampiero Baroncelli, Rosa Pasquariello, Enrico Bertini, Giovanni Cioni

**Affiliations:** Department of Developmental Neuroscience, IRCCS Stella Maris, Viale del Tirreno 331, 56128, Calambrone Pisa, Italy; Department of Obstetrics, Gynecology, and Pediatrics, Pediatric Division, Santa Chiara University Hospital, Pisa, Italy; Laboratory of Molecular Medicine, Ospedale Bambino Gesù Research Chidren’s Hospital, Rome, Italy; Department of Clinical and Experimental Medicine, University of Pisa, Pisa, Italy

**Keywords:** 4H leukodystrophy, *POLR3B* gene, Hypomyelination, Hypogonadotropic hypogonadism, Growth impairment, Panhypopituitarism, Recombinant GH

## Abstract

**Background:**

The 4H syndrome (hypomyelination, hypodontia, hypogonadotropic hypogonadism) is a newly recognized leukodystrophy. The classical form is characterized by the association of hypomyelination, abnormal dentition, and hypogonadotropic hypogonadism, but the recent identification of two genes (*POLR3A* and *POLR3B*) responsible for the syndrome demonstrates that these three main characteristics can be variably combined among “Pol-III (polymerase III)-related leukodystrophies.”

**Case presentation:**

We report on the clinical, neuroradiological and endocrinological follow-up of a male affected by 4H syndrome with confirmed *POLR3B* mutations (c.1568 T > A/p.V523E variant in exon 15 and the novel c.1988C > T/p.T663I mutation in exon 19). Spastic-ataxic gait with worsening of motor performance, progressive moderate intellectual disability and language difficulties were the main neurological findings observed. The first six years of substantial stability of the clinical and imaging features were followed by additional six years that showed a progressive worsening of motor, language and learning disabilities in relation to a progression of the cerebellar involvement. Hypogonadotropic hypogonadism and growth hormone deficiency followed by central hypocortisolism became part of the patient’s phenotype. Thyroid function resulted unaffected during follow up.

**Conclusions:**

A novel mutation in *POLR3B* in a patient with an analogue phenotype than those previously described but with more extensive endocrinological features, including hypogonadotropic hypogonadism, growth hormone deficiency and hypocortisolism, was described. These findings permit to better define the clinical spectrum of the disease, to direct specific genetic tests and to tailor clinical management.

## Background

Leukodystrophy is a heterogeneous group of inherited neurodegenerative disorders characterized by abnormal central-nervous-system (CNS) white matter at brain imaging [1]. Congenital hypomyelinating disorders are the largest sub-group of leukodystrophies [2]; among these, the 4H syndrome (hypomyelination, hypodontia, hypogonadotropic hypogonadism) (HLD7, OMIM 607694 and HLD8, OMIM 614381) has been recently characterized genetically with manifestations of hypomyelination of the brain and of the peripheral nervous system [1–4], associated, in the classical form, with abnormal dentition (hypodontia) [5] and hypogonadotropic hypogonadism [1–7].

Two causative genes encoding the largest subunits of human RNA polymerase III (Pol III) -*POLR3A* and *POLR3B*- have been identified [8, 9] and mutations in these genes may cause four overlapping hypomyelinating leukodystrophy phenotypes: 1) tremor-ataxia with central hypomyelination or TACH; 2) 4H syndrome; 3) leukodystrophy with oligodontia (LO); 4) diffuse cerebral hypomyelination with cerebellar atrophy and hypoplasia of the corpus callosum (HCAHC) [6–10].

To date, the largest series described consists of 105 patients, of whom 43 have mutations in *POLR3A* gene and 62 in *POLR3B* gene. Except for French Canadian patients, affected individuals from European backgrounds were more likely to have *POLR3B* mutations than other populations [9, 11].

Regarding the imaging studies, Takanashi et al. [12], but also Wolff et al. [11], suggested that the Magnetic Resonance Imaging (MRI) pattern of hypomyelination and cerebellar abnormality may be distinct between patients with *POLR3A* and *3B* mutations. Cerebellar atrophy was found almost in all patients with 4H syndrome [11, 13, 14], but the cerebellar anomalies were more severe in patients with *POLR3B* while the pattern of hypomyelinization was more evident in the MRI of *POLR3A* mutated patients [11, 12].

We report on the longitudinal clinical and MRI study of a new patient with Pol III-related leukodystrophy. The patient was firstly diagnosed as an undefined hypomyelination leukodystrophy and reached the final genetically confirmed diagnosis ten years later, following the description of *POLR3A* and *POLR3B* as disease genes.

Patient’s endocrinological profile in adolescence was evaluated in order to define the endocrine phenotype of this disorder and to give better indications for clinical management.

## Case presentation

### Genotyping

After obtaining informed consent, genomic DNA was extracted from peripheral blood following the manufacturer’s instructions (Qiagen, Hilden, Germany). The entire coding sequence and intron–exon boundaries of *POLR3A* (NM_007055) and *POLR3B* (NM_018082) genes were amplified by PCR using intronic primers designed to flank coding exons. Amplimers were purified using Exo-SAP (GE Healthcare) and directly sequenced using BigDye 3.1 chemistry (Applied Biosystems, Foster City, CA, USA) with an ABI Prism 3130 xl automatic sequencer (Applied Biosystems). Mutations were confirmed in independent reactions by sequencing both strands and segregation analysis of the identified mutations was performed by sequencing the corresponding amplicons in family members.

### Endocrinological methods

Centiles of birth weight and length were calculated according to Italian reference standards [15]; postnatal height was expressed as raw measured values and as standard deviation scores (SDs) according to Tanner et al. [16]. Bone age was assessed according to the method of Greulich and Pyle [17]; mid parental height (MPH) was calculated using measured parental heights adjusted for male sex [(father height + mother height)/2) + 6.5 cm]. Baseline blood samples were obtained in the fasting state between 8.00 and 9.00 a.m.; GH secretion in response to GHRH (1 μg/kg i.v.) plus arginine provocative test (0.5 mg/kg/i.v.) was assessed with sampling at 0, 30, 60, 90, 120 min (normal response = GH peak > 20 ng/ml). Serum levels of LH, FSH, Δ4-androstenedione, testosterone, dihydrotestosterone (DHT), cortisol, and ACTH were measured by commercially kits. All serum samples were kept at −80 °C up till to laboratory assessment. Serum levels of GH and IGF1 (reference values for prepubertal children 277 ± 93 ng/ml) were determined as previously reported [18].

### Neurological examination

At each evaluation a senior neurologist examined the patient using the Brief Ataxia Rating Scale (BARS). This instrument was selected as it could be rapidly administered during the clinical setting without special appliances or equipment; its feasibility and reliability have been well documented in ataxic patients [19].

### Findings

An Italian boy has been referred to our Department at age 6 years for evaluation of developmental delay and because of mild ataxic gait from the beginning of unsupported walking; he was followed overtime until to age 20 years. He was born from an uneventful pregnancy by dystocic delivery [birth weight g 3300 (−0.43 SDS), birth length cm 49 (−0.98 SDS), birth head circumference cm 35 (0.22 SDS)]. His motor and mental developmental milestones were delayed: he was able to walk unsupported at the age of 16 months, however, he was described as “clumsy” and unstable; he spoke with meaningful words at 18 months and brief sentences at 24 months with following speech word by word. Dentition was also delayed: front incisor teething was presented at 4 years and successively normal teeth progression continued. Attention deficit and mild learning impairment were also reported since his primary school attendance.

At the age of 7 years, cerebellum signs as ataxic gait, mild tremor of upper limb, nystagmus, ocular motor abnormalities, such as hypometric saccades, and dysarthric language were the prominent clinical manifestations.

**Fig. 1:**
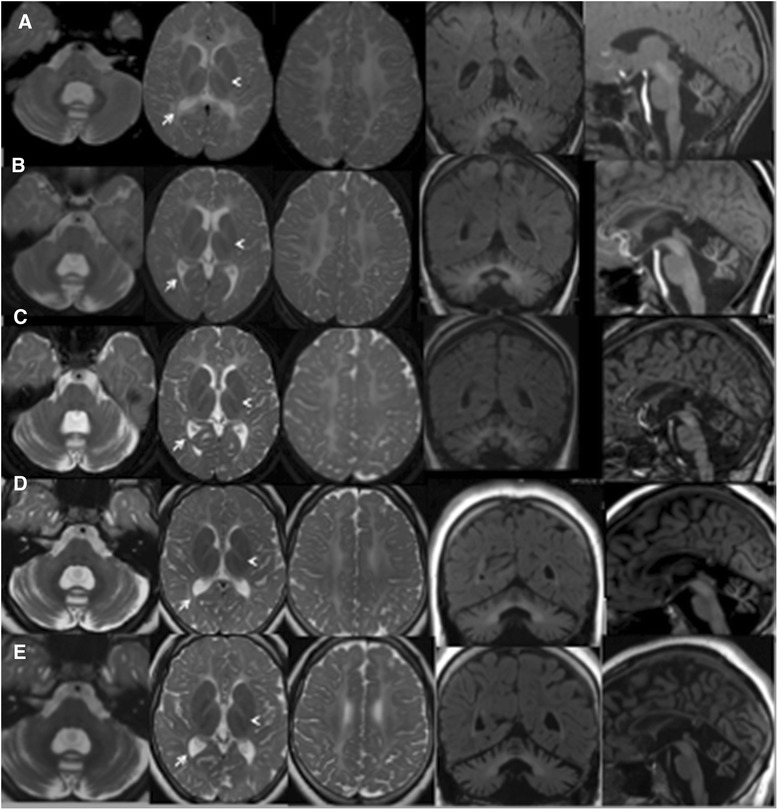
Hypomyelination with cerebellar atrophy: long term follow-up evaluation. **a** (7 years). **b** (10 year). **c** (13 years). **d** (15 years). **e** (19 years): Axial and coronal T2-weighted images show extensive cerebral white matter (WM) abnormalities with predominant involvement of the deep and subcortical WM; note the sparing of optic radiation (arrows), perirolandic WM and partial splenum corpus callosum. The head arrows (**a-e**), instead, indicate small hypointense dot in the posterior limb of the internal capsule. Mild abnormal hyperintensity involves the cerebellar WM. Sagittal T1-weighted images show a thin corpus callosum and shrunken cerebellar cortex with enlarged fissures. The pons is normal. At age of 13 years MRI (c) revealed a mildly increased cortical atrophy of cerebellar hemispheres that remained stable in the following MRI exams. No significative changes were observed during the yrs on WM abnormalies

Brain MRI was characterized by a diffusely hyperintense signal on T2-weighted images of the cerebral white matter with sparing of optic radiation. The cerebellar vermis was atrophic (Fig. 1a).

In order to make a differential diagnosis between the known leukodystrophies, serum levels of aminoacids, alfa-fetoprotein, carcinoembryonic antigen, lysosomal enzyme (arilsulfatase, betaesasominidase, betagalactocerobrosidase), fitanic and pristanic acid, very-long chain fatty acids, sialic acid and isoelectric focussing of transferrin were performed and all of them were within normal limits. In addition, molecular genetics ruled out mutations in *PLP1*, *GJC2/GJA12* and *HSPD1*.

EEG recording showed mild occipital anomalies that disappeared with time, showing only a low amplitude of background activity. Serial electrophysiological studies showed that auditory brain-stem responses (ABR) have always been normal; upper limb somato-sensory evoked potential (SSEP) were abnormal but remained stable with time; nerve velocity conduction showed a sensory peripheral delay, while the motor component was normal. Lower leg motor evoked potentials (MEP) were not detectable while upper limb stimulation was normal.

Flash visual evoked potentials (F-VEP) and electro-retinogram (ERG), instead, showed a slow deterioration with progressive reduced amplitude during the follow-up, consistently the boy developed low visual acuity.

Neurophysiological studies performed during the follow-up are shown in Table 1.

**Table 1:** Neurophysiological data of the patient during follow-up

Age	EEG	ABR	SSEP	mNCV		sNCV		F-VEP/ERG		MEP	
			Upper Limbs (UL)	UL	Lower Limbs (LL)	UL	LL	P100	ERG	UL	LL
7 years	occipital anomalies	N	N9:8.2 ms	N	N	N	N	141 ms	N	----	-----
			N13:9.8 ms					18.7 μV			
			N20:18 ms								
13 years	------	N	N9: 8.6 ms	N	N	50 μV	50 μV	145 ms	N	----	-----
			N13:11.3 ms			2.8 ms	4.3 ms	11.4 μV			
			N20:19 ms			35 m/s	26 m/s				
17 years	Low amplitude background activity	N	-----	----	----	-----	-----	142 ms	N	N	n.d.
								−2.8 μV			
19 years	Low amplitude background activity	N	N9: 8.6 ms	N	N	50 μV	50 μV	n.d.	N	N	n.d.
			N13:13.3 ms			3.1 ms	4.9 ms				
			N20:2 ms			37 m/s	28 m/s				

*EEG* electroencephalogram, *ABR* auditory brainstem response, *SSEP* somatosensitive evoked potentials, *NCV* nerve conduction velocity (m: motor; s: sensitive), *F-VEP* flash visual evoked potentials, *ERG* electroretinogramm, *MEP* motor evoked potentials

-----not done, *N* normal, *n.d* not detectable

Around the age of 10 years intention tremor increased and at age of 13 years, the boy showed progressive gait abnormalities due to spasticity associated with ataxia and slow cognitive regression without discrepancy between verbal and performance quotient (Total IQ 62) became clear. Despite the absence of subjective sensory troubles at lower limbs, the patient showed an electroclinical sensory neuropathy, not reported until now in other patients [3, 11], even if peripheral nerve hypomyelination on electron microscopy in sural nerve biopsy were already described in three patients, despite their normal nerve-conduction studies [3] and a mild lack of myelin was observed also in the nerve biopsy of a patient of Wolf series [11]. These nerve pathology observations seem to be in agreement with the findings on nerve conduction studies of the lower limbs in our patients.

Comparing to the 7 years brain MRI, the important cerebral white matter hyperintensity remained substantially stable in the sequential examinations at 10-13-15 years of age (Fig. 1b, c and d respectively), with the exception of a worsening in the atrophy of cerebellar hemispheres that became evident from age 13 years (Fig. 1c).

At 15 years, the coding region and the flanking exon/intron boundaries of *POLR3A* and/or *POLR3B* genes were amplified and sequenced. Two compound heterozygous missense mutations in *POLR3B* gene were identified: the common V523E variant in exon 15 [9, 11], inherited from his mother and the novel T663I mutation in exon 19, inherited from his father.

At 19 years, the adolescent presented a clear spastic-ataxic gait with worsening of motor performance that included upper limb functions; he could walk autonomously but he needed a wheelchair when walking for long distances.

His cognitive profile worsened, revealing a moderate intellectual disability (Total IQ 32), language became progressively worse and slow and the comprehension of his speech was increasingly difficult.

**Fig. 2:**
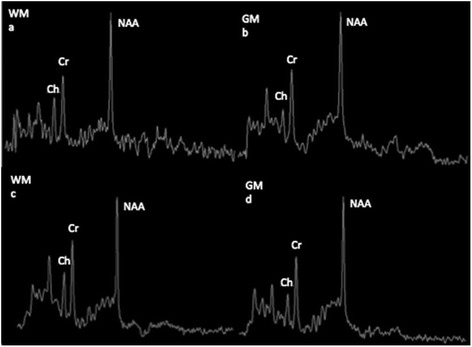
**a-d.** Single voxel short TE (35 ms) 1H-MRS acquired from posterior centrum semiovale (WM) and interhemispheric parieto-occipital gray matter (GM) at 19 years (**a-b**). Single voxel short TE (35 ms) 1H MRS acquired from posterior centrum semiovale (WM) and interhemispheric parieto-occipital (GM) at 19 years (**a - b**); a comparison with the first 1H-MRS acquisition (7 years) was shown (**c-d**). In WM was present a decreased Choline (Cho) that is slightly reduced also in GM; no changes in proton MRS have been observed over the time

Brain MRI showed extensive white matter abnormalities with predominant involvement of the deep and subcortical white matter; the cerebellum was shrunken, with thin folia and enlarged fissures that were moderately abnormal in the hemispheres and severely abnormal in the vermis (Fig. 1e). Proton single voxel Magnetic Resonance Spectroscopy (1H MRS) was added to serial MRI follow-up, acquired from the posterior centrum semiovale white matter and interhemispheric parieto-occipital gray matter. MRS evaluations during the follow-up showed a relative decrease of choline peak, related to the N Acetil Aspartate and Creatine peaks, involving the white matter and slightly the gray matter as well, probably related to the abnormal myelination. These data remained stable during overtime, as demonstrated when comparing between the last examination (Fig. 2a and b) and the first one (Fig. 2c and d).

The neuroradiological pattern of this patient is similar to those reported in patients with *POL3B* gene mutations with a 4H syndrome, particularly related to the cerebellar involvement. Our patient showed, in fact, severe hypomyelination from the beginning of the disease and mild progression of the cerebellar atrophy that was particularly marked in the vermis [2, 13]. As in the large series of patients reported [11] the optic radiation (Fig. 1, arrows) and the dentate nucleus also in our patient were spared; indeed a small hypointense dot in the posterior limb of the internal capsule was seen in our patient too (Fig. 1, head arrows). The last MRI evaluation at 19 years did not show a significative supratentorial atrophy although the cognitive decline could suggest that feature, as reported in literature [11].

The disease progression was very slow at the beginning and progressively accelerated both from a clinical and imaging point of view: in the adolescent age the boy showed worsening of motor disabilities and language, particularly of dysarthria and gait disability, and learning abilities in relation to progression of the cerebellar involvement (Fig. 1a-e).

**Fig. 3:**
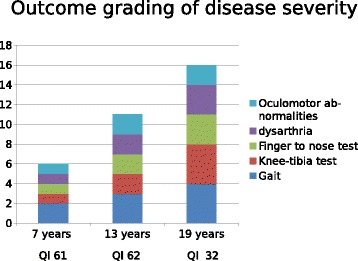
Longitudinal evolution of the disease emerged considering BARS scores (column) and full-scale IQ at Wechsler scale

BARS scale was used to assess the clinical picture and the score of each BARS test designed the longitudinal evolution during the follow-up (Fig. 3). Clinical signs of our patient in association to other outcome measures (Wechsler scale for intellectual quotient and MRI data) were considered to show the outcome grading of disease severity (Fig. 3).

Clinical examination at 19 years showed severe short stature [height, cm 151.0 (−3.6 SDs); MPH, cm: 168.5 (−0.9 SDs)], overweight [kg: 60.5 (weight for height excess: 47.6 %)], and severe puberty delay [G1, Ph2; mean testicular volume, ml: 2 (−8.4 SDs)]. Assessment of reproductive axis was performed and low testosterone concentrations as well as low values of basal and stimulated LH and FSH were found (Table 2), concluding for the diagnosis of hypogonadotropic hypogonadism. Due to height impairment, GH-IGF1 axis was also explored, showing subnormal GH secretion and low IGF1 values, permitting the diagnosis of GH deficiency (GHD). The remaining of the pituitary axis was not affected, but low normal values of both ACTH and cortisol were found (Table 2). Reduced bone mineral density (BMD) was found at both lumbar spine (BMD area: −2.1 SDs; BMD volume −0.9 SDs) and femoral neck (BMD area: −4.6 SDS; BMD volume −5.0 SDs). Treatment with recombinant GH was started (0.25 mg/kg/week), while testosterone administration was delayed to improve the severe growth delay. At 6 and 12 months of GH therapy, growth velocity increased to 7.7 cm/year and to 6.0 cm/year; IGF1 levels normalized (Table 2), but overt central hyposurrenalism was detected after 6 months of GH administration (Table 2) and substitutive treatment with long-acting hydrocortisone (Plenadren^®^, 20 mg/daily) was started. Transdermal testosterone (Tostrex^®^, 10 mg/daily) was added after 12 months of GH administration.

**Table 2:** Endocrinological data at the age 19 years

Parameter	Baseline	6 month GH therapy	Normal values
GH basal, ng/ml	0.3	—	—
GH peak, ng/ml	2.0	—	>9.1
IGF1, ng/ml	84.6	349.0	127-424
LH basal, UI/L	0.6	—	1.4-12.7
LH peak, UI/L	1.1	—	>5
FSH basal, UI/L	1.0	—	1.3 – 19.5
FSH peak, UI/L	1.4	—	—
Testosteone, ng/ml	<0.1	<01	1.7-7.8
ACTH, pg/ml	14	12	<50
Antimüllerian hormone, ng/ml	30.0	—	1.2-15.0
Cortisol, μg/dl^a^	7.0	0.6	6.7-22.6
TSH, μU/ml	1.9	1.8	0.4-3.4
fT3, pg/ml	4.4	4.0	2.7-5.7
fT4, ng/dl	1.2	1.1	0.7-1.7

^a^confirmed in multiple samples in different days and also by reduced values of urinary 24 h cortisol

Thus, hypogonadotropic hypogonadism as a part of the phenotypic spectrum of 4H syndrome was confirmed [4, 6, 20, 21]; however, in our patient, pituitary function resulted more largely affected than usually described, as suggested by the presence of GHD and hyposurrenalism. Normal thyroid function was found in the present adolescent as well as in other patients [11].

GHD has been suspected in another young adult patient, but he showed normal adult stature and dynamic assessment of GH secretion was not performed [20, 22]; thus, diagnosis relied only on low IGF1 values [20]. We explored GH status by a potent test because of a negative impact of adipose tissue on GH secretion [23]. An other adolescent patient with short stature and partial GHD has been described [21] and, recently, additional 5 subjects with 4H syndrome and GHD have been reported in the large series of Wolf et al. [11], but endocrine data to support the diagnosis have been not shown [21]. However, only 10 patients were tested for GHD and the effective number of those with short stature was not shown in the Wolf’s series [11].

Conversely, normal GH-IGF1 axis has been described in an adolescent boy with 4H syndrome and growth delay [6].

In addition, impaired ACTH-cortisol axis developed in this boy in late adolescence. Start of GH therapy may have unmasked a latent hypocortisolism as well known in panhypopituitarism.

Reduced BMD values have been found in the present patient, determining an increase in fracture risk. This is an unreported, but not unexpected, finding of the 4H syndrome, since both GHD and hypogonadism may impair bone health [24, 25]; reduced BMD might be, moreover, related to the poor physical activity, as reported in other neurological diseases [26, 27].

## Conclusions

We report on longitudinal follow-up of Pol III-related leukodystrophy in a boy who manifested the combination of the major clinical findings (hypomyelination, motor dysfunction, abnormal dentition and hypogonadotropic hypogonadism) related to the 4H syndrome [1–7]. The clinical phenotype of our patient shares some features with the four overlapping clinical syndromes described before the identification of the mutations in *POLR3A* and *POLR3B* genes, confirming that the various allelic hypomyelinating disorders do not represent distinct clinical entities but a continuous spectrum. Our patient with early presentation, so far remains ambulant; initial ataxic gait has become progressively spastic; he did not present a true hypodontia but only delayed eruption of his deciduous teeth and a mild intellectual disability with slow cognitive regression, a peripheral neuropathy and multiple pituitary deficiencies associated with low BMD represent additional unusual features, suggesting an intermediate phenotype.

Abnormal smooth pursuit and nystagmus remain the main ocular features without signs of optic atrophy so far that have to be investigated during the follow up.

GHD might be a more common finding than usually considered [11], but the true GH secretory status in patients with 4H syndrome remains unclear till homogeneous and large series of patients will be assessed. Indeed, GH secretory status should be adequately assessed when impaired linear growth is present and GH treatment should be started when GHD was confirmed, considering the efficacy of the GH therapy also at the late age of our subject.

Pituitary pathology may be progressive in the 4H syndrome and hypophyseal function should be monitored during lifespan, to warrant an adequate substitutive therapy patients if pituitary abnormalities will develop, mainly regarding lifesaving therapies as hydrocortisone, because abnormal adrenal function may be implicated in the reduced survival rate reported in these patients [11] in addition to deterioration of neurological functions.

BMD values should be followed during hormonal substitutive therapies to verify its improvement. Regarding bone features, mild osteopetrosis has been reported in 3 patients of Wolf’s series [11], but we did not performed X-ray examination of the skeleton in this patient.

In addition, adequate follow-up should be done during substitutive therapies not only regarding the physical features, but also regarding neurological abnormalities. In fact, experimental data indicated that both GHD and testosterone deficiency may be involved in impairing myelinisation process [28–30].

Our findings expand the clinical spectrum of allelic variants in “Pol-III-related leukodystrophies” and suggest that these mutations are probably under diagnosed in congenital hypomyelinating disorders. For the clinicians, it is important to observe the additional findings of the syndrome to optimize long term management. A better definition of the phenotypic involvement with the aid of a neurophysiologic assessment allows a better understanding of functional deficits and helps in the management of this disorder. In addition, this report supports a progressive hypophyseal endocrine dysfunction with ageing that should be highlighted in larger series of patients.

### Consent

We have obtained the written informed consent from the patient for pubblication of this case report and any accompanying images. A copy of the written consent is available for review from the Editor of this journal.

## Abbreviations

CNS: Central-nervous-system
TACH: Tremor-ataxia with central hypomyelination
LO: Leukodystrophy with oligodontia
HCAHC: Cerebral hypomyelination with cerebellar atrophy and hypoplasia of the corpus callosum
MRI: Magnetic resonance imaging
ABR: Brainstem auditory responses
SSEP: Somatosensory evoked potential
F-VEP: Flash visual evoked potential
ERG: Electroretinogram
IQ: Intellectual Quotient
1H MRS: Proton single voxel magnetic resonance spectroscopy
MPH: Midparental height
GHD: Growth hormon deficiency
BMD: Bone mineral density
SDs: Standard deviation scores
DHT: Dihydrotestosterone
BARS: Brief Ataxia Rating Scale
